# Low Omega-3 Index in Pregnancy Is a Possible Biological Risk Factor for Postpartum Depression

**DOI:** 10.1371/journal.pone.0067617

**Published:** 2013-07-03

**Authors:** Maria Wik Markhus, Siv Skotheim, Ingvild Eide Graff, Livar Frøyland, Hanne Cecilie Braarud, Kjell Morten Stormark, Marian Kjellevold Malde

**Affiliations:** 1 National Institute of Nutrition and Seafood Research (NIFES), Bergen, Norway; 2 Regional Centre for Child and Youth Mental Health and Child Welfare, Uni Health, Uni Research, Bergen, Norway; 3 Department of Biomedicine, Faculty of Medicine and Dentistry, University of Bergen, Bergen, Norway; 4 Department of Clinical Psychology, Faculty of Psychology, University of Bergen, Bergen, Norway; Catholic University of Sacred Heart of Rome, Italy

## Abstract

**Background:**

Depression is a common disorder affecting 10–15% women in the postpartum period. Postpartum depression can disrupt early mother-infant interaction, and constitutes a risk factor for early child development. Recently, attention has been drawn to the hypothesis that a low intake of seafood in pregnancy can be a risk factor for postpartum depression. Seafood is a unique dietary source of the marine omega-3 fatty acids and is a natural part of a healthy balanced diet that is especially important during pregnancy.

**Methods:**

In a community based prospective cohort in a municipality in Western Norway, we investigated both nutritional and psychological risk factors for postpartum depression. The source population was all women who were pregnant within the period November 2009 - June 2011. The fatty acid status in red blood cells was assessed in the 28^th^ gestation week and participants were screened for postpartum depression using the Edinburgh Postnatal Depression Scale (EPDS) three months after delivery. The aim of the present study was to investigate if a low omega-3 index in pregnancy is a possible risk factor for postpartum depression.

**Results:**

In a simple regression model, the omega-3 index was associated with the EPDS score in a nonlinear inverse manner with an R square of 19. Thus, the low omega-3 index explained 19% of the variance in the EPDS score. The DPA content, DHA content, omega-3 index, omega-3/omega-6 ratio, total HUFA score, and the omega-3 HUFA score were all inversely correlated with the EPDS score. The EPDS scores of participants in the lowest omega-3 index quartile were significantly different to the three other omega-3 index quartiles.

**Conclusion:**

In this study population, a low omega-3 index in late pregnancy was associated with higher depression score three months postpartum.

## Introduction

The World Health Organization states that maternal mental health problems pose a huge human, social, and economic burden to women, their infants, their families, and society; and constitute a major public health challenge. Although the overall prevalence of mental disorders is similar in men and women, women’s mental health requires special considerations in view of women’s greater likelihood of suffering from depression and anxiety disorders and the impact of mental health problems on childbearing and childrearing [1].

A number of studies have focused on postpartum depression in women, especially on how the mother’s depressive disorder can affect the infant’s emotional, social, and cognitive development. There has been a debate as to whether postpartum depression differs in aetiology and magnitude from non-postpartum depression [2]. Postpartum depression is prevalent, estimated to affect around 10–15% of all mothers [3], [4] and coincides in time with a period where infants motivation to engage in exchanges of mutual communication of emotional states with their caregivers peaks [5]. A period often referred to as “primary intersubjectivity”. Maternal depression is associated with an impairment in the mother’s ability or motivation to synchronise with the infant’s emotional state [6]. As a consequence, infants of depressed mothers are more likely to express difficulties related to emotional and social regulation during infancy, and difficulties later in childhood, such as cognitive and socio-emotional delay [7]. Moreover, even sub-clinical levels of maternal depression may affect young infants’ expectations of contingent maternal behaviour [8].

A British case-control study of mothers found a three-fold higher incidence of depression within five weeks after childbirth, compared to women who were not pregnant nor had a baby within the past 12 months [9]. Similarly, a Norwegian study comparing postpartum with non-postpartum women found a nearly two-fold greater risk for depression in postpartum women [10]. Whether postpartum depression is a separate form of depression or simply just an important time to identify, it is important to identify factors that are associated with postpartum depression so that prevention is possible. Seen in aetiological terms, depressive disorders are an extremely heterogeneous group and form the final stage of a wide variety of causal pathways [11]. Risk factors for postpartum depression include psychological (history of depression, prenatal depression and anxiety, stressful life events, poor marital relationship and lack of social support), social (low socioeconomic status, marital status, and unplanned/unwanted pregnancy) and biological contributions [12].

Recently, attention has been drawn to the relation between nutrition and mental health. Due to its unique content of important nutrients like marine omega-3 fatty acids, vitamin D and B_12_, iodine, selenium [13], [14], and high-quality proteins [15], consumption fish of and other seafoods has been regarded as an important part of a healthy diet [16]–[22]. The most recent reports from Sweden and FAO/WHO now also emphasise the importance of fish and seafood in relation to mental health and pregnancy [21], [22]. Observational studies and clinical trials have evaluated the possible role of omega-3 fatty acids consumption in the aetiology of postpartum depression and suggest an association between low levels of marine omega-3 fatty acids and the occurrence of depression in the postpartum period [23]–[28]. In a cross-country comparison, Hibbeln found a strong inverse association between fish consumption and prevalence of depressive illnesses [29] and postpartum depression [30]. Moreover, several studies indicate that the dietary intake of omega-3 fatty acids has a large enough impact on brain function to significantly affect mental health [23], [27], [31]–[36].

After fertilisation, the maternal stores of docosahexaenoic acid (DHA, 22∶6n-3) progressively decrease [37], [38], unless the dietary intake from seafood or supplements compensate for the drain to the foetus [39]. As the developing foetus has a high demand for DHA *in utero*, a good marine omega-3 fatty acid status is especially important in fertile women. The general Norwegian recommendation is to eat fish for dinner 2–3 times a week, half of which should be oily fish, and in addition eat fish as spread. Furthermore, women are recommended to continue their fish consumption during pregnancy [40], [41]. Nevertheless, studies have shown that Norwegian women decrease their seafood intake during pregnancy [42]. Other sources of omega-3 PUFAs such as flaxseed oil and rapeseed oil contain α-linoleic acid (ALA, 18∶2n-3) that needs to be converted to longer-chain eicosapentaenoic acid (EPA, 20∶5n-3) and further to DHA to become biologically useful. Consuming omega-3 PUFAs as EPA and DHA is therefore preferable to other sources because the ability of the human body to synthesise EPA and DHA from ALA is limited. A certain, though restricted, conversion of ALA to EPA occurs, however the conversion to DHA is severely restricted. ALA competes with omega-6 PUFAs and uses the same enzymes to be converted to EPA and DHA. Western diets tend to be inadequate in omega-3 PUFAs and rich in omega-6 PUFAs leading to a decreased synthesis of EPA and DHA [43]. Furthermore, there is evidence that fatty acid metabolism could be impaired in some individuals due to for example defects in the fatty acid desaturase gene [44] or oxidative stress in the body [45].

Arachidonic acid (AA, 20∶4n-6) and the marine omega-3 fatty acid, DHA are fundamental components in the brain and in the central nervous system, and play an important role in the growth, development and structure of the brain [46]. EPA has important functions in the synthesis of eicosanoids [47]. Highly unsaturated fatty acids (HUFAs) are mainly esterified into phospholipids and contribute to maintaining membrane fluidity [48]. The primary role of HUFA’s in humans is cell signalling (eicosanoid signalling [49], ion channel modulation [50], regulation of gene transcription, and receptor mediation [51]). Abnormalities in the HUFA composition in cell membranes microstructure, can cause abnormal signal transduction and immunologic dysregulation, and could possibly increase the risk of developing depression [52]. Several studies support uniformity of response to individual HUFA’s, however there are many studies that have shown qualitative and quantitative differences in response to EPA and DHA [51]. An improved understanding of the similarities and differences for EPA and DHA in human metabolism is warranted.

The elucidation of the actual mechanism by which marine omega-3 fatty acids participate in depression is beyond the scope of this article. However, the issue is progressively being studied and an emerging body of research suggest a causal role for these nutrients in the aetiology of depression [53], by linking marine omega-3 fatty acids with efficient neurotransmission and with inflammatory mechanisms connected to depression [52], [54]. Low blood levels of DHA have been found to be associated with postpartum depression [23], [25], [28] and major depression [32], [55], [56], though it is supplementation with EPA that have shown most prominent results when used in the treatment of depression [57], [58]. However, these concepts are not contradictory.

As mentioned above, risk factors for postpartum depression include psychological-, social-, and biological involvement. During pregnancy, some of these factors are more easily effectible than others. A change in diet, for example by introduction of oily fish, is more feasible than a change in socioeconomic status. The hypothesis tested in this study was that a low marine omega-3 fatty acid status in late pregnancy might be a possible risk factor in the multi-factorial aetiology of postpartum depression.

## Materials and Methods

### Design and study population

**Figure 1:**
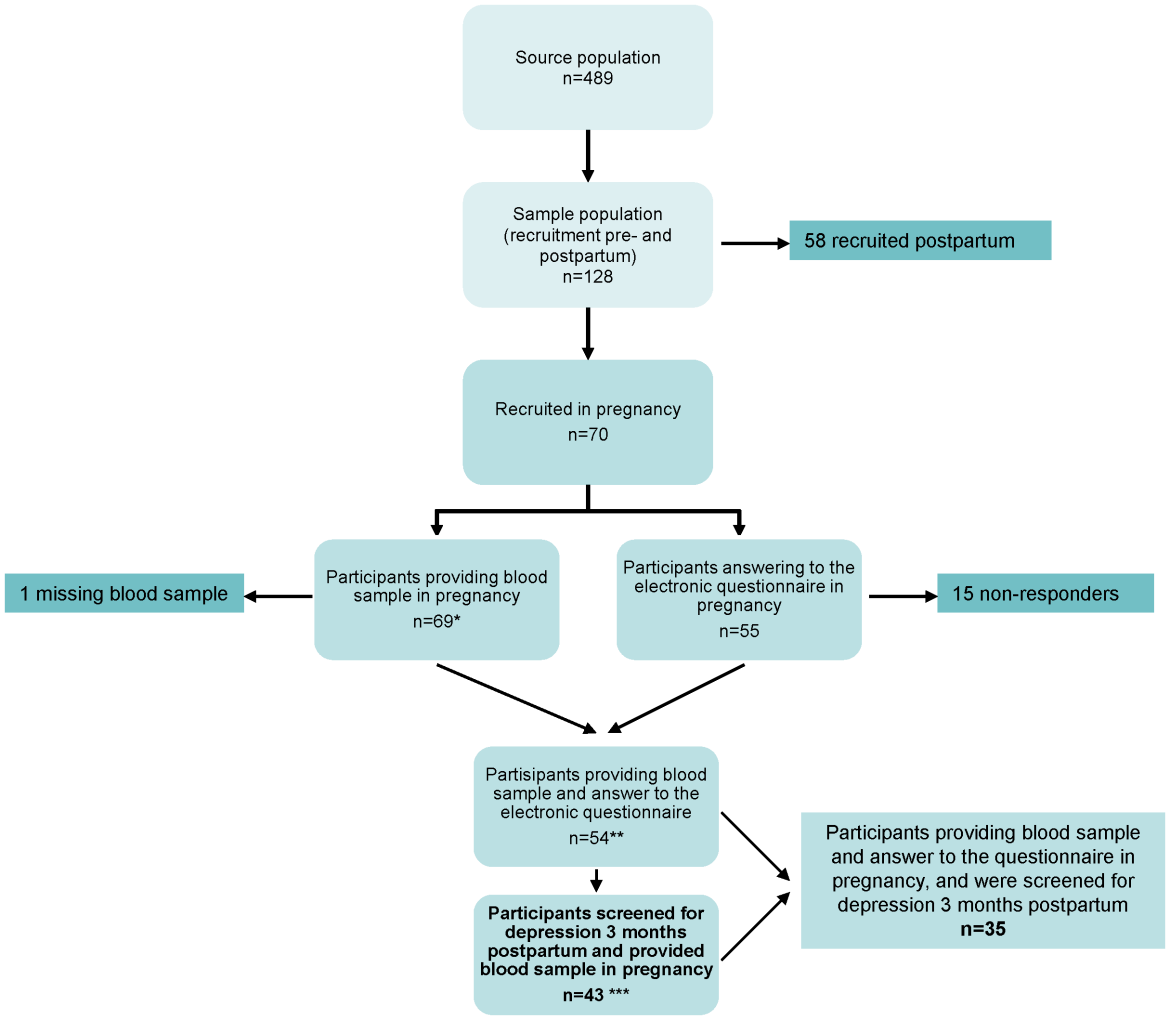
Flow chart of the study design. * Of which one respondent did not provide a blood sample ** Of which 54 responders and 15 non-responders to the electronic questionnaire *** Of which 35 responders and 8 non-responders to the electronic questionnaire. 26 participants were not screened for depression 3 months postpartum due to drop out or losses to follow up.

The investigation originated from a community based study with a prospective cohort design performed in a municipality outside Bergen, Norway. The main objectives were to study the associations between seafood consumption, mental health, and infant development. Enrolment was open for 20 months and the source population was all women pregnant in their 24^th^ week of gestation between November 2009 and June 2011. Midwifes or medical doctors recruited pregnant women at a routine visit in the 24^th^ week of gestation. The original cohort comprised four waves: 28^th^ gestational week, and three, six, and twelve months postpartum. At all time points, a non-fasting venous blood sample was drawn from the participants. The present study includes data from the two first waves. Information regarding other risk factors and potential confounders were collected through a questionnaire sent by e-mail to all participants at the same time points. Figure 1 represents a flow chart of the study design. From the 72 women who enrolled in the cohort, 69 (96%) provided a blood sample for fatty acid analysis in pregnancy, 55 (76%) answered the online questionnaire in pregnancy, and 44 (61%) were screened for postpartum depression at three months postpartum (Figure 1). To reveal if participants that failed to follow-up were a selective group related to the etiological factors under investigation, preliminary analyses were conducted (see Table 1). Women that had provided blood (n = 69) were eligible to further analysis. 43 women (60%) provided blood and were screened for postpartum depression, and were thus eligible for regression analysis. Written informed consent was obtained from all volunteers. Participants could withdraw from the study at any time, without reason. The procedures followed were in accordance with the Helsinki Declaration of 1975 (revised in 2008), and approved by the Regional Committee of Ethics in Medical Research West and the Norwegian Social Science Data Services. A research specific biobank was established and approved for storage of biological samples.

**Table 1:** Socio-economical and behavioural characteristics of study participants (n = 55).

Population characteristics	Count	Percent
**Education**		
Lower secondary school	0	0
Higher secondary school	16	29.1
<4 years of university education†	24	43.6
≥4 years of university education†	15	27.3
**Employment**		
Full-time (80–100%)	48	87.3
Part-time (50–79%)	2	3.6
Part-time (<50%)	1	1.8
Homemaker	1	1.8
Other	3	5.5
**Marital status** ‡		
Married	23	43.3
Cohabiting	28	52.8
Single	2	3.8
**Own income in NOK** §		
<150.000	2	3.6
150.000–199.999	1	1.8
200.000–299.999	12	21.8
300.000–399.999	27	49.1
400.000–499.999	10	18.2
>500.000 NOK	3	5.5
**Self-reported smoking during pregnancy** ‡		
Non-smoker	52	98.2
Current smoker	1	1.8
**Self reported use of snuff during pregnancy** ∥		
Non-user	54	100
Current user	0	0
**Antidepressant use**	1	1.9

† University or university college.

‡ n = 53 (2 participants with missing data).

§ 100 000 NOK ≈ 14 000 EUR.

∥ n = 54 (1 participant missing data).

### Blood Sampling and Fatty Acid Analysis

Venous blood from the elbow cavity was collected in ice water cooled 4 ml BD Vacutainer® K2E 7.2 mg vials for preparation of red blood cells (RBC), and in 3.5 ml BD Vacutainer® SSTTM vials II *Advanced* for preparation of serum. Blood collected in K2E vials was centrifuged (10 min, 1000 g, 20°C) immediately or at least within 30 minutes. RBCs were adequately separated to ensure a clean blood fraction. Blood collected in SSTTM vials was set to coagulate for minimum 30 minutes and maximum 60 minutes prior to centrifugation (10 min, 1000 g, 20°C). Plasma and serum samples were stored at −20°C for 0–4 weeks prior to transportation on dry ice to a −80°C freezer where they were stored until analysis.

The FA composition of total RBC was determined by ultrafast gas chromatographic (UFGC) (Thermo Electron Corporation, Massachusetts, USA), a method developed by Araujo *et al* [59]. After direct methylation of FAs in homogenised samples, boron trifluoride (BF_3_) and internal standard (19∶0 methyl ester) were added, followed by extraction with hexane. The FA composition was calculated using an integrator (Chromeleon 6.80, Dionex Corporation, California, USA), connected to the UFGC and identification ascertained by standard mixtures of methyl esters (Nu-Chek, Minnesota, USA). Limit of quantification was 0.01 mg FA/g samples (wet weight). The analytical quality of the method and systematic errors were controlled by the certified reference materials (CRM) CRM 162 (soy oil) and CRM 163 (pig fat). Results are expressed as relative and absolute amount.

The omega-3 index is the content of EPA and DHA in the RBC membranes, expressed as % of total fatty acids [60]. The omega-3 HUFA score is the % of omega-3 highly unsaturated FA in the total RBC HUFA pool [61] and has been described previously as a biomarker of omega-3 FAs in tissues [62]. In addition to the omega-3 index, the omega-3 HUFA score can also be used as an indicator of disease risk. The omega-3 index was originally suggested as a marker of increased risk for death from coronary heart disease, but it can also be viewed as an actual risk factor, playing a pathophysiologic role in the disease [60], [63]. Optimal levels appear to be 8 or greater. In agreement with Harris et al. we believe that using the Omega-3 index in the design of studies, might allow for a more efficient use of research resources as results from studies will be easier to compare and contrast [64].

### Edinburgh Postnatal Depression Scale (EPDS)

A Norwegian version of the EPDS scale [3], [65] was used to assess the mothers’ level of depressive symptoms, at the regular well-baby check-up three months postpartum. The EPDS was administered by the health care nurse, who followed up the women in need of further assistance. The EPDS is a screening instrument, which consists of ten questions developed to measure the mothers’ depressive feelings during the last seven days. The EPDS-score ranges from 0–30 and a cut-off value of ≥10 is frequently used in primary care setting [3], [65].

### Other Variables and Potential Confounding Factors

A questionnaire designed for the study was sent by e-mail to all participants in the 28^th^ week of gestation. Questions regarding psychosocial aspects like emotional distress, adverse life events, partner satisfaction and social support from family and friends, and socio-demographic aspects, like income, education, marital status and anthropometric measures were included in the questionnaire. Emotional distress in pregnancy was assessed with the Hospital Anxiety and Depression Scales (HADS), a self-reported screening instrument of 14 items designed to detect symptoms of both anxiety and depression [66]. Adverse life events was assessed with a modified version of the Coddingtons list of negative life events [67], used in the Norwegian Mother and Child Cohort Study [68]. Nine adverse life events during the past year were assessed and the items were scored (no = 0 and yes = 1) and summarised. Partner satisfaction was assessed with a modified version of Mehrabians Marital Satisfaction Scale [69] used in the Norwegian Mother and Child Cohort Study [68]. The scale contains 10-items and the overall relationship satisfaction was computed as an average score across items. Social support from family and friends were measured with questions from another Norwegian study [70]. The two scales measuring social support from family and close friends contained 4-items each and the overall level of social support from family and friends were computed as an average score across items.

In the same questionnaire, the seafood consumption was assessed using a semi-quantitative seafood food frequency questionnaire (FFQ). This was designed to capture the habitual intake of seafood and the use of dietary supplements [71] . To enable aggregation and quantity estimation of individual seafood consumption, ordinal data from the seafood-FFQ was converted to numerical data, using the seafood-index system. Details regarding this FFQ and the seafood-index have been explained in detail elsewhere [72].

### Statistical Methods

All statistical analyses were carried out using the Statistical Package for the Social Sciences (IMB® SPSS® Statistics 19, IBM Corporation, Norway). Means (SD) were calculated for the normally distributed variables and median (range) were calculated for the variables that were not normally distributed. Normality of the variables was tested with Kolomogorov-Smirnov statistics. In a simple regression model the omega-3 index (predictor) was associated with the mothers EPDS score (dependent variable). Curve-fitting was applied to obtain the equation that best described the association between the omega-3 index and RBC content of DHA (µg/g) in pregnancy and the motherś EPDS scores postpartum. Pearson product-moment correlation was applied to measure the strength of association between the mothers EPDS scores and all the fatty acids analysed in the final sample size (n = 43, EPDS ≤13).

To investigate the associations between the omega-3 index in pregnancy and other potential risk factors for postpartum depression, and to measure the strength of association between all the measured potential risk factors for postpartum depression and the mothers level of depressive symptoms postpartum, non-parametric correlation analysis were carried out on a smaller sub-sample with available data (n = 35, EPDS≤8). For the comparisons of omega-3 index quartile, the EPDS score in the lowest quartile was tested against the other quartiles applying a Mann Whitney U test. The study was vulnerable to selection bias (non-response bias) as a result of losses to follow-up. People who do not respond (non-responders) usually have different characteristics from those who do respond (responders) [73]. To determine whether participants’ who failed to follow-up were random or systematic, non-responders to the electronic questionnaire were compared to responders, applying a Mann-Whitney U test.

## Results

**Figure 2:**
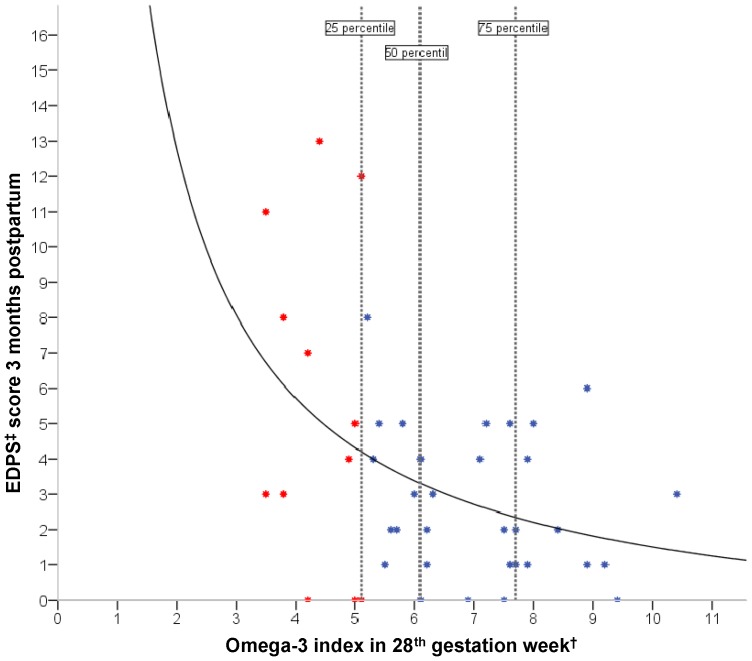
Association between the omega-3 index in pregnancy and postpartum depression. Scatter plot of the non-linear relationship between the marine omega-3 status (omega-3 index) in pregnancy and postpartum depression score (EPDS). A rectangular hyperbolic equation (*y* = -1.31+28.1/x) was the best fit for describing this relationship (*ß* = 0.43, *F* (1,41) = 9.32, *p*<0.004, n = 43). The vertical lines represent omega-3 index quartiles. Red markers represent individuals in the 25 percentile. † The content of EPA+DHA in red blood cells membranes expressed as a percent of total fatty acids ‡ Edinburgh postnatal depression scale.

Lower maternal omega-3 index in late pregnancy (week 28) were related to a higher symptom level of depressive symptoms (*ß* = 0.39, *F*(1, 41) = 7.30, *p*<0.01, n = 43), in a simple linear regression model. An examination of the scatter plot however, suggested that a non-linear curve best described the inverse relationship between the omega-3 index in pregnancy and maternal level of depressive symptoms three months postpartum. The best “goodness of fit” template function was determined by curve fitting in SPSS. This was conducted by selecting the function with the lowest p-value among the highest *R*-squared values. We included linear, inverse (rectangular hyperbola), logarithmic, quadratic and cubic. A rectangular hyperbolic equation (*y* = −1.31+28.1/x) was a better fit for describing this relationship (*ß* = 0.43, *F* (1, 41) = 9.32, *p*<0.01, *n* = 43). The maternal omega-3 index in pregnancy thus explained 19% of the variance in the mothers EPDS scores three months postpartum (Figure 2). Replacing the omega-3 index with DHA (% RBC) in the model resulted in an even stronger nonlinear relationship (*ß* = 0.44, *F = *(1, 41) 10.04, *p*<0.01, *n* = 43).

The mean EPDS score in the study population (n = 43) was 3.5±3.2 and 6.9% of the mothers scored ≥10.

The 25, 50, and 75 percentile of the omega-3 index was 5.1%, 6.1%, and 7.7%, respectively (Figure 2). A Mann-Whitney U Test revealed significant differences in the EPDS scores of participants in the lowest omega-3 index quartile (*Md* = 5.0, *n* = 13) and the three other omega-3 index quartiles (*Md* = 2, *n* = 30), (*U* = 118500, *z* = −2.040, *p*<0.05). The effect size was large (*r* = 0.5) [74].

**Figure 3:**
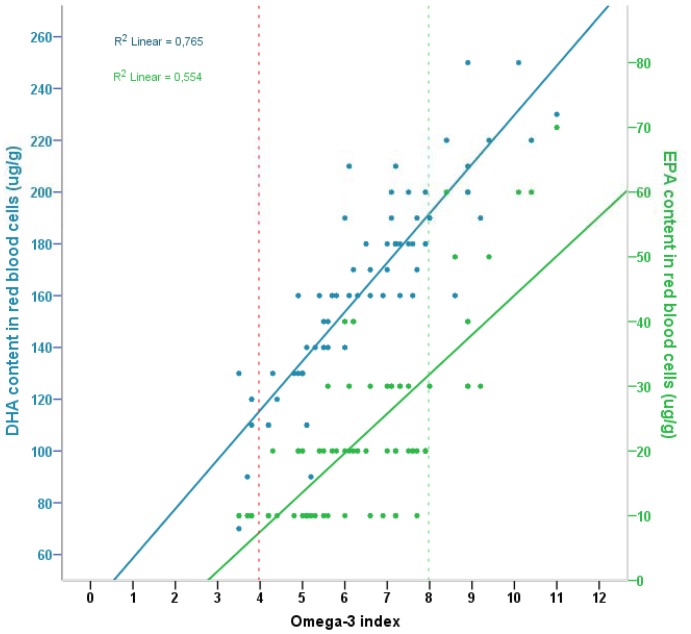
Association between the omega-3 index and absolute DHA and EPA in red blood cells. Scatter plot of the omega-3 index and the corresponding DHA content (µg/g) and EPA content (µg/g) in red blood cells, and the respective fitted regression lines. The omega-3 index is the content of EPA+DHA in red blood cells membranes expressed as a percent of total fatty acids.

There was a strong positive correlation between DHA content (µg/g) in RBC and the omega-3 index (*r* = 0.9, n = 69, *p*<0.001) (Figure 3). We also observed a positive correlation (somewhat weaker) between EPA content (µg/g) in RBC and the omega-3 index (*r* = 0.7, n = 69, *p*<0.001) (Figure 3).

Descriptive characteristics of the study population, obtained from the electronic questionnaire (n = 55) is presented in Table 1. The mean age and BMI of the participants were 29±5 years and 24±3.9 kg/m^2^, respectively.

Participants reported consuming seafood for dinner 1.2±0.5 times per week and seafood as spread 1.1±1.2 times per week. Omega-3 supplements were consumed on average 4.9±3.0 times per week. The correlation between seafood and omega-3 supplement intakes, expressed as the total seafood index, was significantly correlated with the omega-3 index (*r_s_* = 0.34, *p* (one-tailed) <0.01). The correlation between seafood bread spread from fatty fish (>5 g fat/100 g) was likewise (*r_s_* = 0.34, *p* (one-tailed) <0.01).

Descriptive statistics (mean ± standard deviation) of the fatty acid profile (expressed as relative and absolute amount) in the study population, and Pearson’s correlation coefficients between the relative amounts of fatty acids in RBC, in pregnancy, and the mothers EPDS score three months postpartum, is presented in Table 2. RBC DPA status (relative and absolute content), RBC DHA status (relative and absolute content), the omega-3 index, the RBC omega-3/omega-6 ratio, the RBC total HUFA score, and the RBC omega-3 HUFA score, were all inversely correlated with the EPDS score. RBC EPA (relative and absolute amount) did not have a significant correlation with the EPDS score.

**Table 2:** Fatty acid profile of the study population, and Pearson’s correlation coefficients between the fatty acids in red blood cells, expressed as relative amount, and the EPDS‡ score (n = 43).

Fatty acid	Fatty acid (mean ± SD) expressed as:		Pearson’s	*p-value*
	% of total fatty acid	µg fatty acid	correlation§	
		per g red blood cell		
14∶0	0.6±0.2	18±10	0.14	0.38
16∶0	23.2±1.5	666±119	0.25	0.10
18∶0	13.9±1.9	391±33	−0.09	0.56
20∶0	0.3±0.1	<10	†	†
24∶0	0.2±0.3	<10	†	†
*Σ Saturated fatty acids*	*39.2±2.5*	*1119±140*	*0.12*	*0.43*
Σ 16∶1	1.0±0.5	30±21	0.12	0.43
Σ 18∶1	15.8±1.6	457±111	0.26	0.09
Σ 20∶1	0.2±0.2	<10	−0.01	0.98
24∶1n-9	1.7±0.4	47±10	−0.23	0.14
*Σ Monounsaturated fatty acids*	*18.7±2.0*	*540±131*	*0.20*	*0.20*
18∶2n-6 Linoleic acid	13.0±2.9	382±142	0.27	0.08
18∶3n-6	0.1±0.0	<10	†	†
20∶2n-6	0.3±0.1	<10	†	†
20∶3n-6	1.7±0.4	<10	†	†
20∶4n-6 Arachidonic acid	11.2±1.7	320±51	−0.20	0.21
22∶4n-6	2.0±0.5	55±13	−0.06	0.71
22∶5n-6	0.4±0.2	12±4	0.10	0.52
18∶3n-3 α-Linolenic acid	0.3±0.1	11±4	0.08	0.59
20∶5n-3 Eicosapentaenoic acid (EPA)	0.7±0.4	21±13	−0.26	0.10
**22∶5n-3 Docosapentaenoic acid (DPA)**	1.7±0.5	49±12	−0.35	0.02
**22∶6n-3 Docosahexaenoic acid (DHA)**	5.7±1.4	160±38	−0.41	0.006
**Omega-3 index^∥^**	6.4±1.7	NA	−0.39	0.01
**n-3/n-6 ratio**	0.3±0.1	NA	−0.31	0.04
**Total HUFA score** ¶	23.6±3.2	NA	−0.35	0.02
**Omega-3 HUFA score** ¶	34.0±6.7	NA	−0.32	0.04

† Measured fatty acid value below the limit of quantification for the method.

‡ Edinburgh postpartum depression scale.

§ Correlation between % of total fatty acid and EPDS.

∥ The content of EPA+DHA in red blood cells membranes expressed as a percent of total fatty acid.

¶ Total HUFA is the sum of the omega-3 and the omega-6 HUFAs, and the red blood cells omega-3 HUFA score equals 100% - omega-6 HUFA.

The correlations between other risk factors for postpartum depression and the mothers omega-3 index in pregnancy, and the correlations between other risk factors for postpartum depression and the mothers EPDS score postpartum (n = 35), are presented in Table 3. The results showed that partner satisfaction (*r_s_* = .48, *p*<.01) were associated with the mothers omega-3 index in pregnancy. In addition, the results showed that only the mother’s level of emotional distress in pregnancy was associated with the mothers EPDS score postpartum (*r_s_* = .39, *p*<.05).

**Table 3:** Spearman’s correlation coefficients (*r_s_*) between background variables/other potential risk factors for PPD† measured in pregnancy, and the omega-3 index‡ in pregnancy, and EPDS§ score postpartum (n = 35).

Background variables and potential risk factors for PPD†	The omega-3 index in pregnancy	EPDS score 3 months postpartum
Age	−.32	−.03
Education level	.25	−.21
Own income	−.06	.01
Emotional distress	−.07	.44*
Negative life events	.06	.21
Partner satisfaction∥	.48*	.09
Social support from friends	.10	.17
Social support from family	.17	.01
Omega-3 index‡	NA	.04

* Significant at *p*<0.01.

† Postpartum depression.

‡ The content of EPA+DHA in red blood cell membranes expressed as a percent of total fatty acids.

§ Edinburgh postpartum depression scale.

∥ n = 33 (two participants with no partner).

**Figure 4:**
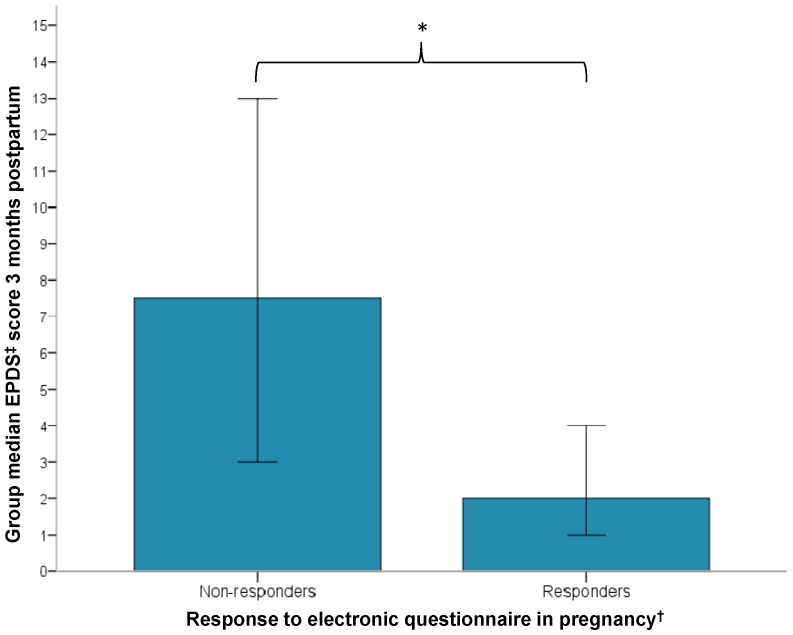
Participant’s response to the electronic questionnaire in pregnancy. Bar chart presenting the median EPDS score measured 3 months postpartum of non-responders (n = 8) and responders (n = 36) to an electronic questionnaire (seafood intake, demography, socioeconomically status, psychological status) in pregnancy. * Significantly difference at the 0.001 level. † 28th week gestation. ‡ Edinburgh postnatal depression scale.

As shown in Figure 4, the comparisons between non-responders (*Md* = 7.5, *n* = 8) and responders to the electronic questionnaire (*Md* = 2.0, *n* = 36), revealed differences in the median EPDS scores, range 0–8 and 3–13, respectively (*U* = 41000, *z* = −3.161, *p*<0.01, *r* = 0.5). The participants who did not answer the questionnaire had a significantly higher EPDS score compared to those who did answer the questionnaire.

Table 4 shows comparisons of the marine omega-3 fatty acid status between non-responders (n = 8) and responders (n = 35) to the online questionnaire, who were screened for depression postpartum. All measured biomarkers of marine omega-3 fatty acid status were significantly lower in non-responders compared to responders. The same results were found between non-responders (n = 15) and responders (n = 54) to the online questionnaire, regardless of whether they were screened for depression postpartum or not.

**Table 4:** Comparisons of the marine omega-3 fatty acid status between non-responders (n = 8) and responders (n = 35) to the online questionnaire.

Marine omega-3 biomarker	Responders†	Non-responders
	Mean ± SD	Mean ± SD
EPA (µg/g RBC)	22±14	11±4*
DPA(µg/g RBC)	51±12	40±9*
DHA (µg/g RBC)	169±36	124±23**
Omega-3 index‡	6.8±1.6	4.6±0.8**
Omega-3 HUFA score§ (RBC)	36±6	27±3**

† Participants who answered questionnaire and provided blood sample for fatty acid analysis, n = 35.

‡ The content of EPA+DHA in red blood cells membranes expressed as a percent of total fatty acid.

§ Total HUFA is the sum of the omega-3 and the omega-6 HUFAs, and the red blood cells omega-3 HUFA score equals 100% - omega-6 HUFA.

* Significantly difference between groups by Man Whitney U test (*P*<0.05).

** Significantly difference between groups by Man Whitney U test (*P*<0.001).

## Discussion

In the present study we found that lower maternal omega-3 index in late pregnancy (week 28) was associated with higher levels of depressive symptoms postpartum, in a simple nonlinear regression model. The curve that best described the association was a rectangular, hyperbolic relationship. The kink in the curve was at an omega-3 index of 5.1%, which was also the 25-percentile level in the study population. Thus, we suggest that maternal marine omega-3 status in pregnancy could be a possible biological risk factor for postpartum depression. The nature of the association was in concurrence with the findings of Jacka *et al*. who recently found a nonlinear relationship between dietary intakes of DHA and depressive disorders in women [75]. A nonlinear relationship between a low omega-3 index and depression is also supported by other studies [30], [76]. The nonlinearity could indicate that the risk factor first becomes active as a possible aetiological factor for depression at a certain low level. In the present study, this level was at an omega-3 index of about 5%, with prevalence in the study population of 25%. An omega-3 index of 5.1% corresponded to about 130 µg/g DHA in RBC. In the target population, the prevalence of an omega-3 index <5.1% might be over 25% taking into account the homogeneity of our study population, where both education and economic status were right skewed. An omega-3 index >5.1% protecting against depression resembles the pathophysiologic picture seen in cardiovascular disease, where a low omega-3 index is associated with an increased risk for death from coronary heart disease [60], [64]. In cardiac health an omega-3 Index of ≥8% is associated with the greatest cardio protection, whereas an index of ≤4% is associated with the least [60]. The omega-3 index appropriate for reducing risk for depression is not known. However, the present study aids the concept of the omega-3 index into the field of mental health, as suggested by Milte and colleagues [77].

**Figure 5:**
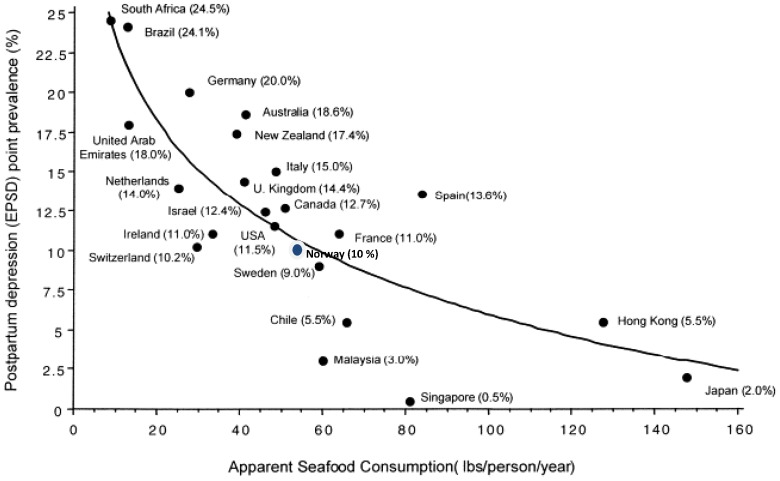
Seafood consumption and prevalence rates of postpartum depression. Postpartum prevalence rates for Australia, New Zealand, Sweden and the United Kingdom, The United States were derived by meta-analysis. All other countries are represented by a single study, see text. Apparent Seafood consumption lb/person/year is an economic measure of disappearance of all fish and seafood from the economy and is calculated by imports plus catch minus exports. A logarithmic regression was used for analysis (*r* = −0.81, *p*<0.001). In Norway the seafood consumption is (47 lbs/person/year = 21,3 kg/person/year [94] and the prevalence of postpartum depression is 10% [3]. Modified from Hibbeln *et al.* [30].

When studying seafood consumption and postpartum depression, Hibbeln *et al.* [30] also found a non-linear inverse relationship (Figure 5). The measures are however not identical to the current study. Hibbeln *et al*. applied prevalence rates of EPDS scores in different countries related to the apparent seafood consumption (an economic measure of disappearance of all fish and seafood from the economy calculated by imports plus catch minus exports) in the same countries. In the current study, individual EPDS scores as the dependent variable were related to the omega-3 indexes as the independent variable in the same individual. The resemblance of Figure 2 (results from the current study) and Figure 5 (results from Hibbeln *et al.* [30]) is prominent. Thus, the non-linear inverse relationship between apparent seafood consumption and postpartum depression on a population level was strengthened in the current study based on individual omega-3 indexes and EPDS scores.

Participants in the study population reported consuming seafood for dinner 1.2±0.5 times per week and seafood as bread spread 1.1±1.2 times per week. Omega-3 supplements were consumed on average 4.9±3.0 times per week. The correlation between seafood and omega-3 supplement intakes, expressed as the total seafood index, was significantly correlated with the omega-3 index. Likewise, the correlation between seafood bread spread from fatty fish (>5 g fat/100 g) and the omega-3 index. Further details concerning the seafood consumption of the same study population (n = 54) has been published elsewhere [72]. Participants with a low omega-3 index had a low seafood and/or omega-3 supplement intake. A low seafood and/or omega-3 supplement intake (*total* seafood index, 0≤4) were associated with a lower EPA (µg/g RBC) and DHA (µg/g RBC) status than those with a higher seafood and/or omega-3 supplement intake (*total* seafood index, >8–11).

To our knowledge, this is the first study to investigate the omega-3 index in late pregnancy as a possible risk factor for postpartum depression measured 3 months after delivery. However, Pottala *et al.* [36] recently found that adolescent depression was associated with a reduced omega-3 index. The results from the present study are supported by other studies. Rocha and Kac [78] found that the prevalence of postpartum depression was 2.5 greater among Brazilian women whose dietary ration of omega-6/omega-3 in the first trimester was greater than 9∶1, as they would be expected to have a low omega-3 index in the 28^th^ gestation week. The findings in our study are also in somewhat concurrence with the findings in a Belgian observational study where women who became depressed after delivery had a significantly lower DHA status and total omega-3 FA status in their phospholipids and cholesteryl esters in serum, shortly after delivery, compared to non-depressed controls [23]. Theoretically, one would assume that mothers who have a low omega-3 index in pregnancy who also have a stable habitual diet, could lead to an even lower omega-3 index postpartum due to foetal accretion of PUFAs and nursing [79]. Our findings is also supported by the results of Otto et al. who found that a slower normalisation after pregnancy in the functional DHA status (DHA/DPA ratio) was associated by an increased risk of depressive symptoms in the postpartum period [80].

When studying pregnant Canadian women with a high level of depressive symptoms during mid-pregnancy in relation to fish consumption and intake of omega-3 HUFAs, Sontop *et al*. [81] found an association between low dietary intakes of EPA and DHA and more depressive symptoms. However, the association was only found among current smokers and women of single marital status, indicating that a low omega-3 status is only a risk factor for postpartum depression in this sub-group of women. The study had a large sample size (n = 2394) and 18.8% of the participants scored above ≥16, the cut-off commonly used to indicate probable depression using the Centre for Epidemiologic Studies-Depression Scale. Nevertheless, the median dietary intake of EPA and DHA was only 85.1 mg/day and a dichotomised variable based on the median was used as a predictor in the multiple regression analysis. Dietary recommendations for EPA and DHA, set by the European Food Safety Authorities is 250 mg/day for primary prevention of cardiovascular risk in healthy people [82]. The dietary EPA and DHA intake in Sontop *et al*.’s study was also extremely skewed with little variation, indicate that the study was conducted in a population with a very low daily intake of EPA and DHA. In such population, an association between prenatal depression and the intake of EPA and DHA, as a possible bio etiological risk factor, is not expected, especially not if the association between omega-3 intake and mental health is nonlinear [30], [76]. Results from the large Danish National Birth Cohort showed little evidence in support of an association between intake of fish or omega-3 HUFAs and postpartum depression [83]. The study had a large sample size (n = 54202) and a median DHA intake of 310 mg daily for the third quintile of the study population [84]. However, depression was measured by admission to the hospital for postpartum depression (0.3% of participants) or prescription of antidepressants within one year postpartum (1.6% of participants). The authors acknowledged that the measure of depression was a limitation to the study.

To our knowledge, data from the Avon Longitudinal Study of Parents and Children have the most representative population to study the association between seafood intake and maternal depression. They found that at a lower maternal intake of omega-3 from seafood was associated with high levels of depressive symptoms in late pregnancy (n = 9960) [85]. Compared to women who consumed more than 1.5 grams of omega-3 fatty acids from seafood per week (≈215 mg/day), those consuming none were more likely (adjusted OR = 1.54; 95% CI = 1.25–1.89) to have high levels of depressive symptoms at 32 week’s gestation.

A few randomised interventions studies, using supplements of DHA, found no association between DHA supplement intake and depressive symptoms in the postpartum period [86]–[88]. It is important to note that none of the three studies referred to measure the omega-3 status (in the body) at any time during the trial, only the intake of omega-3 fatty acids in form of supplements was measured. In addition, the intervention trials differ a great deal and the results are therefore difficult to evaluate. In order to compare the findings from different studies, it is important that the timeframe and length of the study are strictly considered.

There are to date no established reference values for fatty acid status in pregnant or lactating women. Such values are warranted. In the current study, we correlated the omega-3 index with both the absolute amount of DHA (μ/g RBC) and EPA (μ/g RBC). As expected, there was a significant positive correlation between the individual fatty acids and the omega-3 index. The association was stronger between DHA and the omega-3 index than between EPA and the omega-3 index. Based on the non-linear negative association found between the maternal omega-3 status in pregnancy and the mothers level of depressive symptoms postpartum in the current study we have postulated that omega-3 index as a possible risk factor first becomes active as an aetiological factor for postpartum depression at a level of about 5%. In reference to the regression line between DHA (µg/g RBC) and the omega-3 index, a DHA status above 130 µg DHA/g RBC might be suggested as preferable in relation to maternal mental health. Low levels of the fatty acid in RBC make a subsequent value more difficult to suggest for EPA.

Risk factors associated with postpartum depression can be separated into psychological, social, and biological characteristics [12], [89] and it can be difficult to distinguish between these risk factors. Some factors may be more directly related to the condition, while others as causes of the condition. In a multi factorial aetiological picture, it can be difficult to adjust for confounding factors that might bias the result. Sometimes adjusting for a factor that is not a real confounder, might introduce a bias where none existed.

With studies that require commitment over time, retention of participants is a common challenge. In this community-based study, only 58% were eligible for a regression analysis as they provided data on both the predictor and outcome variable. Others have noted that characteristics of women least likely to complete studies of prenatal care include younger, lower education, lower income, and/or higher parity [90], [91]. Our study was in concurrence to other studies where higher levels of depression has been associated with participant attrition [73]. Participants who were lost to follow-up in the current study were systematic concerning the aetiological factor under investigation. Compared to responders, women who did not respond to the questionnaire sent by e-mail had a higher mean EPDS score and a lower mean omega-3 index. Consequently, a distinct limitation to this study was that it was not possible to control for the effect of confounders, as this group of participants did not provide data concerning any potential confounders. A selective drop-out effect or missing data could have significant implications if the selection is according to the outcome variable or there is a non-linear relationship between predictor and criterion [92], both of which are appropriate in the current study. However, in order to address this limitation, a correlation analysis was conducted on the available sample in order to investigate if the mothers omega-3 status in pregnancy where associated with any of the other potential risk factors for depressive symptoms. The available sample for this analysis included data from those participants that had provided both answer to the electronic questionnaire and a blood sample for fatty acid analysis in pregnancy.

The mother’s omega-3 index correlated significantly with the mother’s satisfaction with partner relationship. The more satisfied they were with their partner, the higher their omega-3 index. However, the mother’s satisfaction with partner relationship was not related to the mother’s level of depressive symptoms postpartum. Theoretically, partner satisfaction could act as confounders in our simple regression model. However, it is unlikely to believe that this factor act as confounder for the nonlinear association found between the omega-3 index in pregnancy and the mothers level of depressive symptoms postpartum.

Only emotional distress during pregnancy was associated with the mothers’ level of depressive symptoms in the sub-sample where both biological and psychosocial data were available (EPDS ≤8). In this sub-sample none of the other risk factors, including the mother’s omega-3 status in pregnancy, where associated with the mothers level of depressive symptoms postpartum. The finding that emotional distress during pregnancy is related to maternal level of depressive symptoms postpartum accords with earlier findings [4], [14], [93]. Bringing this together, one possible interpretation is that that the mother’s level of emotional distress during pregnancy is related to the mothers level of depressive symptoms postpartum, even in population-based samples, but that the maternal omega-3 status in pregnancy first operates as a risk factor for postpartum depression at a certain low level.

Adequately powered intervention studies and longitudinal observational studies that are larger in size, and that control and adjust for potential confounders in a correct manner, are warranted. However, results from the present study indicate that fertile and pregnant women could benefit from increasing their omega-3 status by increasing their seafood intake, particularly those women that have a nonexistent or low seafood intake, with a subsequent low omega-3 status. For those that for some reason cannot increase their seafood intake, marine omega-3 supplement intake could be introduced. Especially pregnant women who are at risk of developing postpartum depression due to the presence of other known risk factors should have their seafood intake screened, and subsequently be given dietary advice, whilst in antenatal care.
